# Targeted deletion of liver-expressed Choriogenin L results in the production of soft eggs and infertility in medaka, *Oryzias latipes*

**DOI:** 10.1186/s40851-021-00185-9

**Published:** 2022-01-04

**Authors:** Kenji Murata, Masato Kinoshita

**Affiliations:** 1 University of California, Davis. Center for Health and the Environment, Davis, CA 95616 USA; 2 Division of Applied Bioscience, Graduate School of Agriculture, Kyoto University, Kyoto, 606-8502 Japan

**Keywords:** Egg envelope, Chorion, Zona pellucida, Choriogenins, TALEN, Oogenesis, In vitro fertilization, Soft egg, Infertility, Medaka

## Abstract

Egg envelopes (chorions) in medaka, *Oryzias latipes*, are composed of three major glycoproteins: ZI-1, − 2, and − 3. These gene-encoded chorion glycoproteins are expressed in the liver and/or ovarian oocytes of sexually mature female fish. In medaka, the glycoproteins produced in the female liver are induced by estrogen as Choriogenin (Chg.) H and Chg. H minor (m), which correspond to the zona pellucida (ZP) B (ZPB) protein in mammals, and Chg. L, which corresponds to ZPC in mammals. Chg. H, Chg. Hm, and Chg. L, are then converted to ZI-1, − 2, and − 3, respectively, during oogenesis in medaka ovaries.

In the present study, we established a medaka line in which the *chg.l* gene was inactivated using the transcription activator-like effector nuclease (TALEN) technique. Neither intact *chg.l* transcripts nor Chg. L proteins were detected in livers of sexually mature female homozygotes for the mutation (homozygous *chg.l* knockout: *chg.l−/−*). The *chg.l−/−* females spawned string-like materials containing “smashed eggs.” Closer examination revealed the oocytes in the ovaries of *chg.l−/−* females had thin chorions, particularly at the inner layer, despite a normal growth rate. In comparing chorions from normal (*chg.l*+/+) and *chg.l−/−* oocytes, the latter exhibited abnormal architecture in the chorion pore canals through which the oocyte microvilli pass. These microvilli mediate the nutritional exchange between the oocyte and surrounding spaces and promote sperm-egg interactions during fertilization. Thus, following in vitro fertilization, no embryos developed in the artificially inseminated oocytes isolated from *chg.l−/−* ovaries. These results demonstrated that medaka ZI-3 (Chg.L) is the major component of the inner layer of the chorion, as it supports and maintains the oocyte’s structural shape, enabling it to withstand the pressures exerted against the chorion during spawning, and is essential for successful fertilization. Therefore, gene products of oocyte-specific ZP genes that may be expressed in medaka oocytes cannot compensate for the loss Chg. L function to produce offspring for this species.

**Supplementary Information:**

The online version contains supplementary material available at 10.1186/s40851-021-00185-9.

## Background

**Fig. 1:**
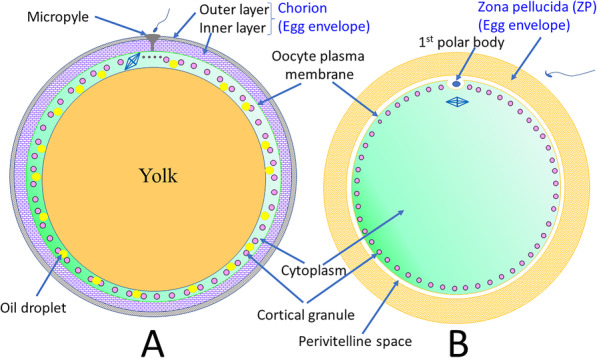
Schematic illustrations of ovulated fish (medaka) and mouse eggs (**A** and **B**, respectively). The fish (medaka) egg envelope, also known as the “chorion,” “zona radiata,” and/or “vitelline envelope,” is shown with its two layers — the inner layer (interna) and the outer layer (externa). The single-layer egg envelope in mammals is termed the “zona pellucida”

In most animals, the egg envelope has several functions, including attraction and activation of spermatozoa, prevention of polyspermy at fertilization, and protection of the developing embryo [1, 2]. In medaka, *Oryzias latipes*, there are two structural parts to the layers of the egg envelope — a thin, high-density outer layer and a thick, low-density, multiple-component inner layer. During oogenesis, the formation of the outer layer begins during the perinuclear oocyte stage. This step is followed by the formation of the inner layer during the previtellogenic oocyte stage [3]. The terminology of the extracellular matrix (egg envelope) surrounding oocytes differs among groups of animals. In fish, it is the chorion, and in mammals, it is called the zona pellucida (ZP [4];; Fig. 1).

The nomenclature of the *ZP* genes and their gene products is confusing because different names have been used for different animal groups. Spargo and Hope [5] classified vertebrate *ZP* genes into four subfamilies: *ZPA, ZPB*, *ZPC*, and *ZPX*. The medaka chorion is comprised primarily of the inner layers which consist of three major glycoproteins — zona pellucida interna (ZI)-1, − 2, and − 3 [6]. Following Spargo and Hope’s nomenclature [5], *ZI-1* and *-2* are classified as *ZPB* genes, while *ZI-3* is a *ZPC* gene.

During the cortical reaction of fertilization, the chorion changes in structure and forms the fertilization membrane. In fish, alveoline [7] and transglutaminase [8–10] are released from cortical granules to promote hardening of the chorion by affecting the cross-linkages between the subunit molecules of the ZI-1, − 2, and- 3 proteins. At hatching, these are the targeted substrates of the hatching enzyme [11].

In 1984, Hamazaki et al. reported that one of the chorion glycoproteins was a spawning-female-specific (SF) substance of extraovarian origin [12]. In 1991, using specific antibodies, Murata et al. discovered, in medaka, other high molecular weight chorion glycoproteins were also produced in the liver of spawning females [13]. These results suggested, in medaka, all major components of the chorion were produced in the liver of spawning females. Thus, the previously discovered SF substance was renamed the low molecular weight SF substance (L-SF), and newly discovered proteins were described as high molecular weight SF substances (H-SF) to avoid confusion. The synthesis of L-SF and H-SF as induced by estrogen (E_2_) in the liver of females and the liver of E_2_-treated males [13–16]. The accumulation of L-SF in the egg envelope of ovarian growing oocytes was also identified after injecting radio-labeled L-SF in the abdominal cavity of mature female medaka [17]. The cDNAs encoding L-SF [18] and H-SF [19] were then cloned from the mature female medaka liver cDNA library, and L-SF and H-SF were renamed Choriogenin L (Chg.L) and Choriogenin H (Chg. H), respectively. Based on their amino acid sequences, Chg. L and Chg. H were determined homologs of mammalian ZPC and ZPB, respectively [18, 19]. In medaka, the genes encoding Chg. L, Chg. H, and Chg. H minor (Chg.Hm) [20] are expressed in the liver of sexually matured females and induced by E_2_. The proteins are then secreted into the bloodstream and transported into the ovary. After modification, Chg. L, Chg. H, and Chg. Hm accumulate and form the 3D structure of the chorion. During the process of accumulation, Chg. H and Chg. Hm are modified to ZI-1 and -2 (ZPB in mammals), respectively, and Chg. L is modified to ZI-3 (ZPC in mammals) in the chorion [21, 22].

Currently, in teleosts, an infraclass of fish that comprises all ray-finned fish apart from early-diverged bichirs, sturgeons, paddlefishes, freshwater garfishes, and bowfins, there are two known sites, the liver and oocyte, of chorion precursor-protein synthesis. The liver-expressed chorion glycoproteins have been cloned in winter flounder [23], medaka [18–20], rainbow trout, and Atlantic salmon [24]. In contrast, the synthesis of the chorion glycoproteins in cyprinids (goldfish, carp, and zebrafish) seems to be restricted to the oocyte [25–28]. Further studies [29–31] have been conducted to understand the phylogenetical relation between fish and other animals. Expression of ZP-related proteins in the ovaries of medaka has been reported [32, 33]. However, the functions of these proteins and whether they are actually components of chorion remain unknown.

As mentioned above, the medaka Chg. L protein is homologous to the mammalian ZPC [18]. The functions of ZPC in mammals are well defined [34], and morphological observations of oocytes from ZPC-loss-of-function transgenic female mice have been reported [35–37]. For example, homozygous mutant *ZPC*^−/−^ mouse follicles have zona-free oocytes (i.e., germinal-vesicle-intact oocytes that lack a ZP matrix), and disorganized coronae radiatae. The corona radiata is the first layer of follicular (granulosa) cells outside the ZP. Despite the lack of a ZP, both ZPA and ZPB, but not ZPC proteins, were detected at the surface of the zona-free oocytes. The zona-free oocytes grew and remained in meiotic arrest before maturation, and the remainder of the follicular structure appeared normal. During folliculogenesis, the zona-free oocytes developed but did not form a proper cumulus-oocyte complex. In general, the null (*ZPC*^−/−^) female mice ovulated, and cumulus masses were detected in the oviduct, but only a few (< 10% of normal) zona-free eggs were recovered. After mating with males proven to be fertile, no zona-free, 2-cell embryos are recovered from *ZPC*^−/−^ females. These null females were never visibly pregnant, and they produced no live offspring.

Recently, genome editing techniques using transcription activator-like effector nuclease (TALEN) restriction enzymes; clustered, regularly interspaced, short palindromic repeat (CRISPR) deoxyribonucleic acid (DNA) sequences; and the CRISPR/CRISPR-associated protein (Cas) system have progressed. Advances in TALEN enzymes [38, 39] and the CRISPR/Cas9 system [40], specifically, have simplified and expedited the creation of gene-targeted medaka strains. In the present study, we created *chg.l−/−* female medaka using TALEN gene-editing techniques to identify the structural and physiological functions of Chg. L (ZI-3) in the chorion surrounding the oocyte during oogenesis and fertilization.

## Methods

### Fish and tissues

In the present study, an orange, Cab [41] variety of medaka and basic procedures described by Murata and Kinoshita [38] were used to establish a parental (F0), *chg.l−/−* (knockout; KO) medaka line.

All fish were maintained in an aquarium, with recirculating water, under a 14-h (h)/10-h day/night cycle at 26 °C, at small animal facilities, at the Center for Health and Environment, University of California, Davis and Kyoto University, Japan, following the required animal protocols at each university (University of California, Davis Approval No. 2020649; Kyoto University, Japan Approval No. 27–45). Approximately ten wild-type (*chg.l +/+*) female fish were randomly selected as controls from the breeding tank. All fish samples were obtained from F1 or later-generation fish whose genotypes were confirmed as heterozygous or homozygous for the *chg.l* mutation using sequencing or the Heteroduplex Mobility Assay (HMA; 38).

### Antibody production

The potential peptides for antibody production were searched using epitope analysis resources (http://www.iedb.org/). The amino acid sequences of the peptides used for antibody production are shown in Supplemental Table S1. The antigen peptides and antibodies were produced in GenScript USA Inc. (Piscataway, NJ 08854, USA).

### Design and construction of TALENs

**Fig. 2:**
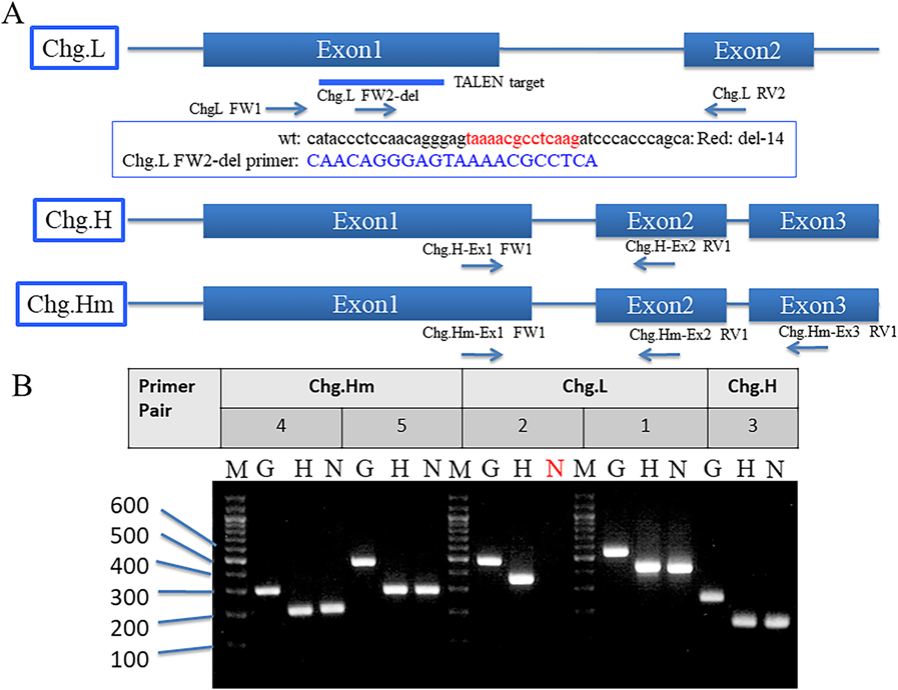
RT-PCR analysis of *chg.l+/*+, *chg.l+/−*, and *chg.l−/−* medaka. **A** The position of the primers used for the analysis. **B** The result of RT-PCR analysis of *chg.l+/−* and *chg.l−/−* medaka. Abbreviations: M – marker; G – genomic DNA extracted from the *chg.l+/+* fin with sodium hydroxide; H – *chg.l+/−* (del-14 heterozygous knockout (KO)); N – *chg.l−/−* (del-14 homozygous KO). The targeted genes for the RT-PCR assay, primers, size of the cDNA product, and anticipated size of the genomic DNA are summarized in Supplemental Table S2. The primer Pairs for PCR analysis included (1) Chg. L FW1 and Chg. L RV2, (2) Chg. L FW2-del and Chg. L RV2, (3) Chg. H Ex1 FW1 and Chg. H Ex2 RV1, (4) Chg. Hm Ex1 FW1 and Chg. Hm Ex2 RV1, and (5) Chg. Hm Ex1 FW1 and Chg. Hm Ex3 RV1

Using TALE-NT 2.0 (https://tale-nt.cac.cornell.edu/) software [42], the potential TALEN target sites were searched with parameters including (1) a spacer length of 14–17, (2) a repeat array length of 15–18, and (3) an upstream base of T only. TAL effector repeats were assembled as described by Ansai et al. [40]. The TALENs were designed in Exon1 of *chg.l* (NM_001104803), with 15 base pairs (bp) of the left binding site (5′- CATACCCTCCAACAG -3′), 15 bp of the right site (5′-AGATCCCACCCAGCA-3′), and a 16-bp spacer sequence (5′-ggagtaaaacgcctca-3′; Fig. 2A and Supplemental Table S2).

### RNA preparation and microinjection

The TALEN expression vectors were linearized using digestion with *Not* I. Capped ribonucleic acid (RNA) sequences were synthesized using the mMessage mMachine SP6 kit (Life Technologies, Gaithersburg, MD). The transcribed RNAs were purified using the RNeasy Mini kit (Qiagen, Valencia, CA) according to its RNA-clean-up protocol. Purified RNAs were diluted with Yamamoto’s Ringer’s Solution (0.75% sodium chloride (NaCl), 0.02% potassium chloride (KCl), 0.02% calcium chloride (CaCl_2_), and 0.002% sodium bicarbonate (NaHCO_3_); pH = 7.3) to a final concentration of 300 ng/uL [43] and microinjected into fertilized eggs.

### Extraction, genotyping, and sequencing of genomic DNA from embryos, larvae, and adult females

The embryos without a chorion (i.e., chorion-free embryos), hatched larvae, and caudal fin clips from adult fish were lysed individually in 25 μL of alkaline lysis buffer (25 mM sodium hydroxide (NaOH) and 0.2 mM of ethylene diamine (EDTA); pH = 8.0) at 95 °C for 10 min (min). Once neutralized with 25 μL of 40 mM Tris–hydrochloric acid (Tris-HCl; pH = 8.0), the tissue lysates were used as genomic DNA samples. The primer pair for genotyping was Chg. L Fw1 and Chg. L RV1. The sequence and genomic position of each primer are summarized in Fig. 2 and Supplemental Table S1. During genotyping, the thermal cycler was held at 94 °C for a 2 min denaturing step followed by 35 cycles of (1) 98 °C for 10 s (sec) for additional denaturing, (2) 60 °C for 10 s of annealing, and (3) 68 °C for 15 s of extension using KOD-FX DNA polymerase (TOYOBO, Osaka, Japan). The resulting polymerase chain reaction (PCR) product was directly sequenced by Eurofins Genomics (Tokyo, Japan) using the Sanger method.

### Reverse transcription polymerase chain reaction (RT-PCR) of liver tissue RNA from adult females

RNA was extracted from the liver of *chg.l+/+* (normal, wild-type control), *chg.l+/−*, and *chg.l−/−* (KO) females using PureLink RNA Mini kits (Thermo Fisher Scientific, Tokyo). The cDNAs were obtained using SuperScript VILO™ Master Mix (Thermo Fisher Scientific, Tokyo) with thermal cycler conditions at 25 °C for 10 min, 42 °C for 60 min, and 85 °C for 5 min. The targeted cDNA fragments were then amplified using KOD-FX (Toyobo, Osaka) with PCR conditions described in the previous paragraph. The primers pairs for this PCR assay were (1) Chg. L Fw1 and Chg. L RV1, (2) Chg. L Fw2-del and Chg. L RV2, (3) Chg. H Ex1 FW1 and Chg. H Ex2 RV1, (4) Chg. Hm Ex1 FW1 and Chg. Hm Ex2 RV1, and (5) Chg. Hm Ex1 FW1 and Chg. Hm Ex3 RV1.

### Preparation of liver and ovary tissue extracts for sodium-dodecyl-sulfate polyacrylamide gel electrophoresis (SDS-PAGE)

Tissue extracts were prepared following the procedures described by Murata et al. [13, 15, 16] after cutting the tail and collecting the blood sample by bleeding the fish from the caudalis (caudal aorta and caudal vein). Briefly, for each fish, the liver and ovary were each dissected, transferred into a 1.5-ml tube containing 100 μl of Tris-buffered saline (TBS) containing 40 mM of EDTA, and homogenized. After centrifugation at 14000 rpm for 10 min at 4 °C, the supernatant was used as the tissue extract. Immediately after preparation of the tissue extract, SDS-sample buffer containing 2-mercaptoethanol was added at a 1:1 ratio with the extract. The diluted extracts were then boiled for 5 min and stored at -20 °C before use in the SDS-PAGE assay.

### Western blot analysis of liver and ovary tissue extracts

SDS-PAGE and Western blot analyses were performed following the methods described by Murata et al. [13, 15, 16]. The proteins in the SDS-PAGE samples were separated using 8% and/or 10% SDS-PAGE gels. After each SDS-PAGE assay was performed, the proteins were transferred onto a polyvinylidene fluoride **(**PVDF) membrane (Immobilon-P; MILLIPORE Co. Billerica, MA, USA). The proteins on the membrane were stained with Coomassie Brilliant Blue R-250 (CBB), and the SDS-PAGE patterns of the tissue extracts obtained from the *chg.l−/−* and *chg.l+/+* females were compared. After treatment with 1:2000-diluted anti-Chg.L and anti-Chg.H antibodies, and 1:5000-diluted secondary antibodies [horse radish peroxidase (HRP)-conjugated goat anti-rabbit immunoglobulin G (IgG; A16096; Invitrogen, Waltham MA USA) and HRP-conjugated rabbit anti-mouse IgG (62–6520: Thermo Fisher Scientific, Waltham MA USA), respectively], the immunoreactive protein bands were visualized using a TMB substrate kit (Vector Lab. Inc. Burlingame. CA).

### Immunohistochemistry (IHC)

Ovaries were collected from *chg.l+/+* (control) and *chg.l−/−* females after the aforementioned bleeding step. Ovary samples were pre-fixed with 4% paraformaldehyde (PFA) in 0.1 M phosphate buffer (pH = 7.2) at 4 ^o^ C for 48 h. After rinsing with TBS, the ovaries were soaked with ice-cold 100% methanol and stored at -20 °C for future use in IHC assays. The paraformaldehyde/methanol-fixed tissues were processed following procedures described by Murata et al. [19]. Briefly, each paraffinized ovary obtained from a *chg.l−/−* female was cut to 5-μm thickness using a microtome to maintain the structure of the oocytes during the deparaffinization and rehydration processes required for staining with IHC antibodies. The sections were deparaffinized using a xylene and alcohol series and rinsed three times with TBS for 20 min. The sections were then incubated with primary anti-Chg.L antibodies, diluted 1000 times with 2% bovine serum albumin (BSA)-TBS containing 0.05% Tween-20 and 2% pre-immune goat serum (BTTBSG), and left overnight at 4 °C. Over the next three consecutive days, the sections were rinsed with TBS and incubated overnight at 4 °C with goat anti-rabbit Alexa 488 antibodies (Invitrogen, Carlsbad, CA 92008 USA), mouse anti-Chg.H primary antibodies, or goat anti-mouse Alexa 568 antibodies (Invitrogen, Carlsbad, CA 92008 USA), each diluted 1000 times with BTTBSG, respectively. After the final incubation, the sections were rinsed with TBS, mounted on glass slides in mounting medium (90% glycerol, 10% of 50 mM Tris-HCl containing 0.15 M NaCl (TBS; pH = 7.4) with 50 mM N-propyl gallate) and coverslipped with glass.

### Immunofluorescence imaging

The sections stained with antibodies as above were observed using an Olympus Fluoview 500 confocal laser scanning microscope mounted on an Olympus BX61 upright fixed-stage microscope (Olympus Imaging America Inc.), each equipped with fluorescence water immersion objectives.

### Electron microscopic observations

To elucidate the chorion structures of the ovarian oocytes from *chg.l+/+* and *chg.l−/−* females in detail, transmission electron microscopic (TEM) observations were performed. The TEM procedures described by Murata et al. [44] were followed. Briefly, survey-thick (about 500 nm) sections of the chorion were cut with a diamond knife (Diatome, Hatfield, PA) and used to produce ultra-thin (about 90-nm) sections from selected areas of the chorion. The ultra-thin sections were placed on copper grids, and the grids were stained with uranyl acetate and lead citrate before viewing via TEM (Philips CM120 Biotwin Lens, F.E.I. Company, Hillsboro, OR, U.S.A). The micrographs were taken with a Gatan MegaScan, model 794/20, digital camera (Pleasanton, CA) and used to measure the thickness of the chorion in *chg.l+/+* and *chg.l−/−* oocytes. Fifteen different portions of the chorion in the *chg.l+/+* and *chg.l−/−* oocytes were randomly selected. The measurements of chorion thickness were analyzed by a student paired t-test (t-Test Calculator: https://www.graphpad.com/quickcalcs/ttest1.cfm) and summarized in Supplemental Fig. S1.

### Observation of mating and spawning behavior of *chg.L −/−* females

The *chg.l−/−* females and normal *chg.l+/+* males were separated the night before mating and held overnight in a dark laboratory. Early the next morning, immediately after illuminating the laboratory, the *chg.l−/−* females and normal males were placed in a single tank and their mating and spawning behaviors were observed and recorded using a charge-coupled device (CCD) camera.

### Collection of ovulated and fully grown pre-ovulated oocytes, and in vitro fertilization

The following procedures were taken to observe whether the oocytes produced in the ovaries of *chg.l−/−* females ovulate and to identify the function of Chg. L in fertilization.

### Preparation of oocytes

The ovaries were dissected from the *chg.l−/−* and control females 2 h before the lights in the facility were turned on, at the time the fish were expected to ovulate. Female fish were anesthetized with 0.03% of tricaine methanesulfonate (MS-222). Briefly, each fish was placed on a culture dish (9 cm in diameter), and the abdominal cavity was opened under a stereomicroscope using fine forceps and scissors. The ovaries were gently removed, immersed in a culture dish (3 cm in diameter), and filled with Leibovitz’s L-15 Medium (Thermo Fisher, Vacaville, CA) to allow avulsion of the ovarian epithelia with fine forceps to release ovulated oocytes. The germinal epithelia of the ovary were carefully peeled off using scissors and forceps after the ovulated eggs were obtained, and the fully grown pre-ovulated oocytes were scattered in a culture dish filled with Leibovitz’s L-15 Medium.

### Preparation of sperm

Once each mature male medaka was anesthetized**,** scissors were used to open the abdominal wall from anus to gill along the dorsal peritoneal cavity. The wall and the dorsal peritoneum were then separated using a pair of forceps to isolate and surgically remove a testis. The peritoneum and fat were removed, and the testis was placed in a 3-cm diameter culture dish.

### Fertilization

The testis was placed into the culture dish containing the aforementioned ovulated and/or pre-ovulated oocytes in Leibovitz’s L-15 Medium. The medium was then removed and replaced with 1 mL of Embryo Culture Medium (ECM: 0.1% NaCl, 0.003% KCl, 0.004% CaCl_2_*2H_2_O, and 0.016% MgSO_4_*7H_2_O), and the testis was minced with a pair of sharp forceps to release the sperm and initiate fertilization. The microscope-mounted CCD camera was used to observe the cortical reaction and movement of oil droplets in the artificially fertilized egg during fertilization and later, during embryonic development.

## Results

### Establishment of the *chg.L −/−* strain

A pair of TALENs targeted for Exon 1 of the *chg.l* gene (Fig. 2A) was injected into 50 fertilized medaka eggs, and 12 eggs hatched. Five females possessing gonads with a frame-shifted genome were mated with a wild-type Cab male, thereby producing an F1 generation. When these F1 fish reached adulthood, DNA sequencing of the targeted region was performed from a piece of each tailfin. More than two types of insertion or deletion (in/del) mutations were observed in F1 fish derived from the five founder-females. Among the F1 fish with mutations, two pairs of F1 mutants, with the paired male and female both exhibiting 7 (del-7) or 14 (del-14) base deletions on the target site, respectively, were selected, and their offspring were bred to establish the *chg.l*−/− medaka strain. Only the results obtained from del-14 KO fish are shown. No phenotypic differences were observed between *chg.l +/+* and *chg.l−/−* males. Figure 2A shows the position of the primers used to confirm the mutated sequence containing the TALEN target region by direct sequencing of the PCR product obtained from the KO larva. Figure 2B shows the combination of primers used to detect the targeted PCR product and the result of the RT-PCR assay. DNA was not amplified with the RNA extracted from the *chg.l*−/− larva using the primers designed from the sequence containing the TALEN-targeted sequence (Fig. 2B, red letter N). However, it was amplified using the primers designed from the DNA sequence outside the targeted sequence (Supplemental Table S2). The smaller size DNA was amplified from reverse-transcribed DNA obtained from the KO larva and compared with that obtained from the normal larva.

Direct sequencings were performed to confirm the mutated sequence in the TALEN target region. The results revealed that the 14-bp nucleotide sequence was deleted (data not shown). In this strain, the reading frame was shifted, resulting in the formation of a truncated Chg. L protein instead of the mature Chg. L (Supplement Table S3) exhibited in the wild type. In fact, to maintain the strain and obtain the null fish, the heterozygous (*chg.l +/−*) mutant fish were bred because the null (*chg.l −/−*) mutant could not successfully spawn eggs.

### Spawning of the *chg.L−/−* females: the ovaries and ovulated eggs

**Fig. 3:**
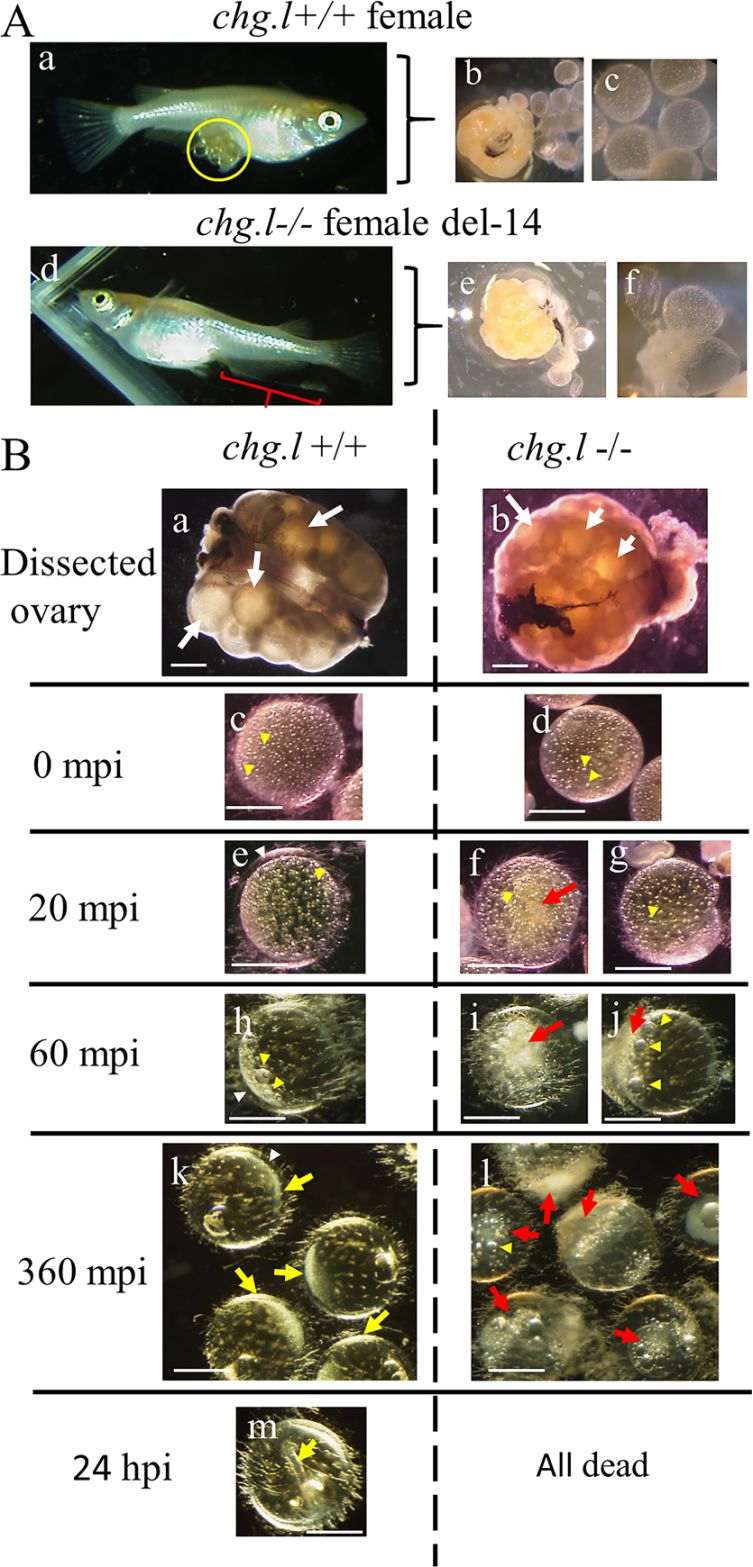
Spawning females, ovaries, and oocytes of *chg.l+/+* and *chg.l−/−* medaka. Panel **A** Spawning females, dissected ovaries, and ovulated eggs of *chg.l+/+* and *chg.l−/−* medaka. **Aa** and **Ab** *chg.l+/+* and *chg.l−/−* females, respectively, with a yellow circle highlighting spawned eggs in from the former and a red bracket highlighting spawned string-like materials containing “smashed eggs” from the latter. **Ac** and **Ad** Isolated ovary and ovulated eggs from the *chg.l+/+* female. **Ae** and **Af** Isolated ovary and ovulated eggs from the *chg.l−/−* female. Panel **B** Changes in the artificially inseminated eggs. **Ba** and **Bb** *chg.l +/+* and *chg.l −/−* ovaries, respectively, used to obtain ovulated and fully-grown oocytes (white arrows) for this “*in vitro* fertilization” experiment. **Bc** and **Bd** *chg.l+/+* and *chg.l−/−* eggs, respectively, immediately before insemination. **Be** and **Bf-g** *chg.l*+/+ and *chg.l*−/− eggs, respectively, at 20 min post-insemination (mpi). **Bh** and **Bi-j** *chg.l+/+* and *chg.l−/−* eggs, respectively, at 60 mpi, **Bk** and **Bl** *chg.l+/+* and *chg.l−/−* eggs, respectively, at 360 mpi, **Bm** *chg.l+/+* eggs at 120 h post-insemination (hpi). White arrowheads show the perivitelline space in **Be**, **Bh**, and **Bk**. Yellow arrowheads show oil droplets in **Bc-h** and **Bj**. Yellow arrows show developing embryos in **Bk** and **Bm**. Red arrows show white turbidity, such as emulsions in **Bf**, **Bi**, and **Bl**. Scale bars in **Aa** and **Ab** = 1 cm; in **Ac-f** and **Ba-b** = 1 mm; and in **Bc-l** = 5 mm

The mating and spawning behavior of *chg.l−/−* females was similar to those of normal females. However, the *chg.l−/−* females did not spawn normal eggs. Instead, string-like material containing smashed eggs was observed extruding from the genital pore (Fig. 3Ab). On the day after spawning, the abdominal cavity of each female was opened surgically to dissect the ovary and obtain ovulated oocytes. The sizes and shapes of the ovaries from *chg.l−/−* (Fig. 3Ae) and *chg.l+/+* (Fig. 3Ac) females were compared and found to be similar. However, most of the ovulated *chg.l−/−* oocytes were smashed, and only a few oocytes with a spherical shape were detected. The *chg.l−/−* oocytes (Fig. 3Af) were softer than those found in *chg.l+/+* females (Fig. 3Ad) and easily smashed when held gently with forceps.

### In vitro fertilization

In vitro fertilization was performed using ovulated oocytes (post-vitellogenic oocyte stage X [3]) and fully-grown pre-ovulated oocytes (post-vitellogenic oocyte stage IX [3]) from *chg.l+/+* and *chg.l−/−* females to identify the function of Chg. L in fertilization. Ovaries were dissected from *chg.l+/+* and *chg.l−/−* females and found to contain both ovulated oocytes (Fig. 3Ac-d and Ae-f, respectively) and fully-grown pre-ovulated oocytes (white arrows in Fig. 3Ba-b), both of which were collected for in vitro fertilization experiments. The ovarian sacs were carefully removed and ovulated, and the fully grown pre-ovulated oocytes were carefully isolated to avoid structural damage to the oocytes, particularly in the *chg.l−/−* ovary. The oocytes isolated from *chg.l−/−* females (white arrows in Fig. 3Bb) were used for the in vitro fertilization experiments.

As the controls, ovulated eggs (Fig. 3Ac-d) and artificially collected fully-grown pre-ovulated oocytes from the *chg.l+/+* ovary (Fig. 3Ba white arrows) inseminated with sperm obtained from sexually mature male medaka resulted in normal embryo development (Fig. 3Bm). In inseminated *chg.l−/−* eggs, the cortical granule and oil droplet movements that occur in normal fertilized eggs (Fig. 3Bc, Be, and Bh) were observed after fertilization (Fig. 3Bd, Bf-g, and Bj). However, the movement and coalescence of oil droplets did not occur as expected (Fig. 3Bi, Bj and Bl) when compared with inseminated eggs from the control (Fig. 3Bh and Bk). Rather, the perivitelline space was not developed, embryonic development in the inseminated oocytes from the *chg.l−/−* females was not observed, and the inside of the eggs became cloudy as all of the fertilized eggs died (red allows in Fig. 3Bf, Bi, and Bl) after 360 min post insemination (mpi).

### Morphological comparison and TEM observation of ovarian oocytes from *chg.L+/+* and *chg.L−/−* females

**Fig. 4:**
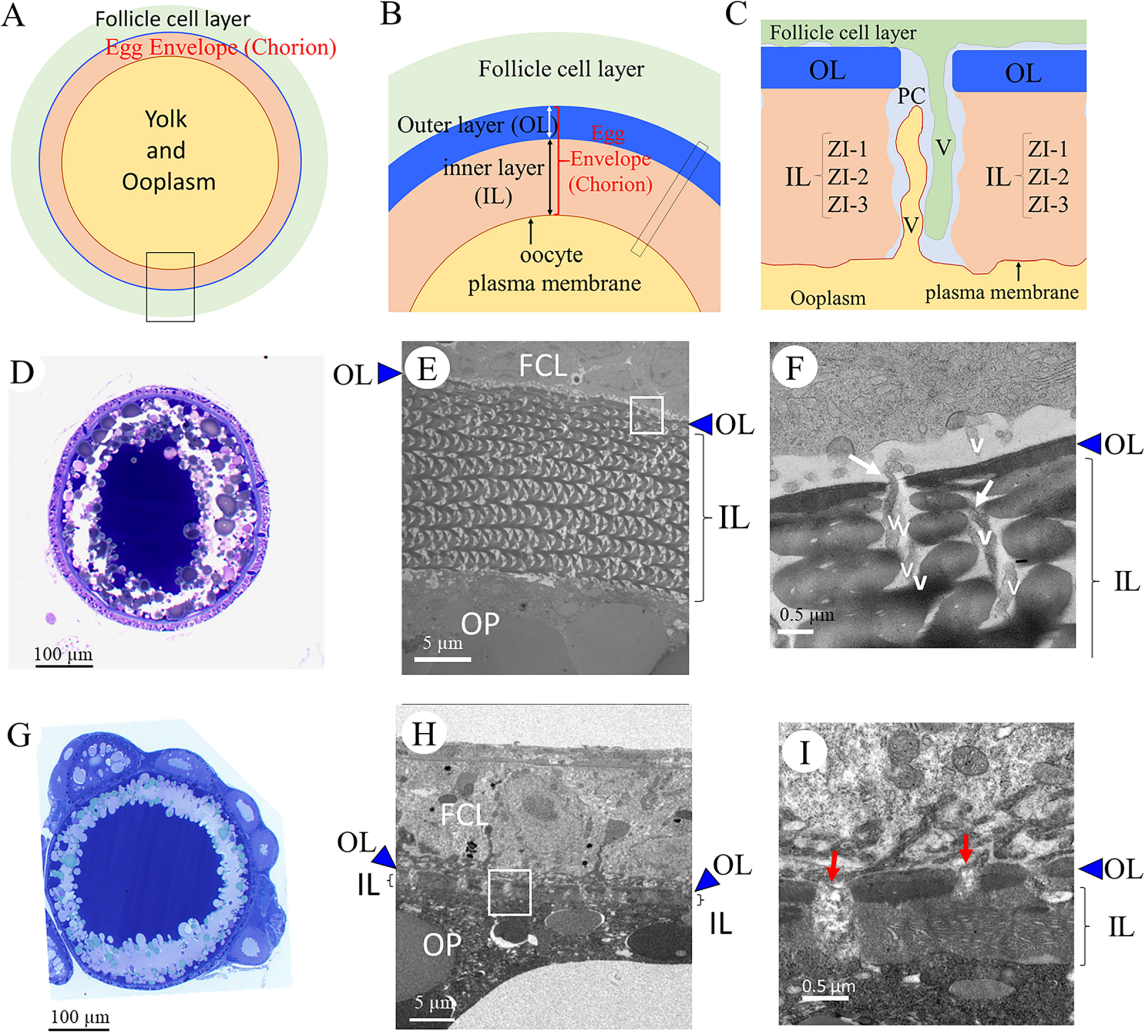
Morphological characterization of ovarian oocytes in the *chg.l+/+* and *chg.l−/−* females. **A-C** Schematic illustrations of the structure of the ovarian *chg.l+/+* medaka egg under different magnifications. **B** Enlarged illustration at the position of the rectangle in **A. C** Enlarged illustration at the position of the rectangle in B, showing microvilli (v) from the follicle cell layer (FCL), ooplasm, and pore canal (PC). **D-F** Light micrograph (**D**) and Transmission Electron micrographs (**E-F**) of the chorion in the *chg.l+/+* female. **G-I** Light micrograph of the ovarian oocyte (**G**) and Transmission Electron micrographs (**H-I**) of the chorion in the *chg.l−/−* female. **F** and **I** Magnified images of the chorion marked by white squares in **E** and **H**, respectively. Symbols – Panels **E-F** and **H-I** OL and dark blue arrowheads with a black outline mark the position of the chorion externa. IL and brackets mark the position of the chorion interna. Panel **F** White arrows mark the position of the pore canal. Panel **I** Red arrows mark large pore canal-like structures. Scale bars in **D** and **G** = 100 μm; in **E** and **H** = 5 μm; and in **F** and **I** = 0.5 μm

TEM observations were performed to identify the *chg.l−/−* chorion structures. The morphology of the ovarian oocyte was quite normal except for the thickness of the chorion (Fig. 4).

In general, the interna of the *chg.l−/−* chorion was thinner (less than 1/10 the thickness) when compared with that at the same developmental stage of *chg.l+/+* oocytes (Fig. 4H-I, E-F, and Supplemental Fig. S1). The thickness and structure of the externa of the chorion were similar among the two types of oocytes (dark blue arrowheads with a black outer line in Fig. 4E-F and H-I). However, the pore canals in the *chg.l−/−* chorions appeared crushed, leaving a reduced space for the microvilli to pass through the follicle cell and into the oocytes. Aside from this, abnormally large pore canal-shaped structures that penetrated from the follicle cell layer to the inside of the oocyte existed in the chorion (red arrows in Fig. 4I).

### Immunoblotting analysis of ovary and liver extracts obtained from sexually mature *chg.L+/+* and *chg.L−/−* females

**Fig. 5:**
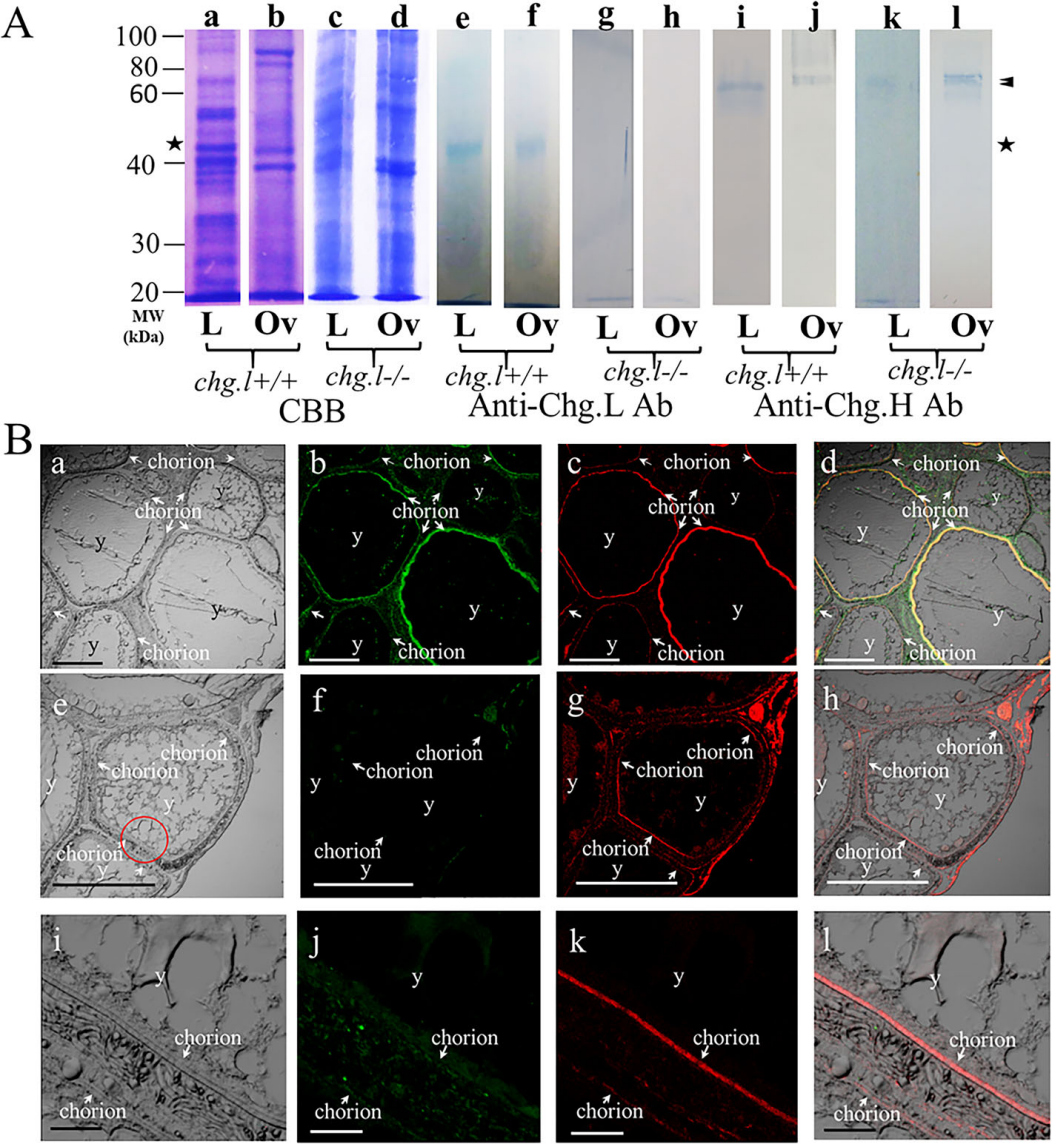
Western blot analysis and IHC detection of egg envelope proteins in the ovaries of *chg.l+/+* and *chg.l−/−* females using specific antibodies against Chgs. Panel **A** SDS-PAGE results showing proteins stained with CBB (**Aa-Ad**) or immunoreactive proteins with anti-medaka Chg. L (**Ae-Ah**) or Chg. H (**Ai-Al**). The numbers on the left indicate the molecular weights of the proteins (blue bands) in kilodaltons (kDa). Symbols - Arrowheasds: the position of Chg. H and Chg. Hm (corresponding to ZI-1 and -2 as chorion components). Stars: the position of Chg. L (corresponding to ZI-3 as a chorion component). No immunoreactive proteins were observed in the negative controls for the Western blot assay (i.e., with primary antibody omitted; data not shown) or the experiment (i.e., the liver and blood plasma extracts from male fish; data not shown). Abbreviations - L: liver extracts. Ov: ovary extracts. Panel **B** Immunohistochemical detection of egg envelope proteins in the ovaries of the *chg.l+/+* and *chg.l−/−* females with anti-Chg.L and anti-Chg. H antibodies. **Ba**, **Be**, and **Bi** Transmission light micrograph of the observed ovarian oocyte in the ovary of the c*hg.l+/+* female (**Ba**), ovarian egg in the ovary of the c*hg.l−/−* female (**Be**), and magnified portion in **Be** circled with red (**Bi**). **Bb**, **Bf**, and **Bj** Immunoreactivity with anti-Chg.L antibodies in the same ovarian oocyte as shown in **Ba**, **Be**, and **Bi**, respectively. **Bc**, **Bg**, and **Bk** Immunoreactivity with anti-Chg.H antibodies in the same ovarian oocyte as **Ba**, **Be**, and **Bi**, respectively. **Bd** Transmission light micrograph (**Ba**) overlaid with **Bb** and **Bc**. **Bh** Transmission light micrograph (**Be**) overlaid with **Bf** and **Bg**. **Bl** Transmission light micrograph (**Bi**) overlaid with **Bj** and **Bk**. Symbol - White arrows: position of the chorion. Abbreviation - y: yolk. Scale bars represent 500 μm (**Ba**, **Bb**, **Bc**, **Bd**, **Be**, **Bf**, **Bg**, **Bh**) and 50 μm (**Bi**, **Bj**, **Bk**, **Bl**)

Figure 5A shows the immunoreactivity of the protein extracts obtained from the liver and ovary of *chg.l+/+* (Fig. 5Aa-b, Ae-f, and Ai-j) and *chg.l−/−* (Fig. 5Ac-d, Ag-h, and Ak-l) females against anti-Chg.L and anti-Chg.H antibodies. The proteins were also stained with CBB (Fig. 5Aa-d). The staining patterns observed for the liver (Fig. 5Aa and Ac) and ovary (5Ab and 5Ad) extracts were similar for the two genotypes, but the protein assumed to be Chg. L from immunoblot results (Fig. 5) was not detected in extracts from *chg.l−/−* tissues (Fig. 5Ac-d). The extracts of mature males were analyzed as negative controls; however, no immunoreactive proteins were detected with the two antibodies (Data not shown.). As shown in Fig. 5Ae and Af, the anti-Chg.L antibody reacted only with 50-kilodalton (kDa) proteins in the liver and ovary extracts obtained from *chg.l+/+* females. It did not react with any proteins in the extracts obtained from *chg.l−/−* females.

Figure 5Ai-Al show the immunoreactive proteins of the liver and ovary extracts against the anti-Chg.H antibodies. The anti-Chg.H antibodies reacted with 74- to 76-kDa proteins in the extracts obtained from *chg.l+/+* and *chg.l−/−* females (Fig. 5Ai-j and Ak-l, respectively). The results obtained from the *chg.l+/*+ females showed the anti-Chg.L and anti-Chg.H antibodies reacted with Choriogenin L and Choriogenin H, respectively. The immunoreactivities of the samples tested here against the anti-Chg.Hm antibodies were the same as those tested against the anti-Chg.H antibodies (Data not shown). These results suggested the presence of Chg. H, but not Chg. L, in the *chg.l−/−* female tissue extracts.

### IHC analysis of Chg. H and Chg. L in the chorions of sexually mature *chg.L+/+* and *chg.L−/−* females

As shown in Fig. 5B, the anti-Chg.L and anti-Chg.H antibodies clearly bound to the chorion in the ovarian oocytes (Fig. 5B) of *chg.l+/+*, but not *chg.l−/−*, females. This result strongly suggested that Chg. L was not present as a component of the chorion in *chg.l−/−* ovarian oocytes. Because faint signals were detected in the *chg.l−/−* chorion with the anti-Chg.H antibodies, a portion of the chorion in Fig. 5Bc (red circle) was magnified and observed. As shown in Fig. 5Bi-Bl, the immunoreactive proteins in the *chg.l−/−* chorion were detected using only the anti-Chg.H, and not the anti-Chg.L, antibodies.

## Discussion

As a first step to identifying the function of Chg. H, Chg. Hm, and Chg. L during oogenesis and fertilization, a *chg.l−/−* medaka transgenic line was established using the TALEN technique. The phenotype and behavior of the *chg.l−/−* fish were normal relative to *chg.l+/+* fish [45, 46] except for the chorions surrounding the oocytes. As shown in Fig. 3Ae and Af, the ovaries in *chg.l−/−* females contained ovulatable oocytes (i.e., oocytes mature enough to be ovulated), but they were easily smashed when held gently with forceps. Observations made during spawning revealed the *chg.l−/−* females extruded string-like material containing smashed eggs from the genital duct (red bracket in Fig. 3Ab).

### Chg.L is essential for oogenesis

In teleosts, ovarian development has been classified as synchronous or asynchronous according to the growth pattern of the oocyte in the ovary at any one time [43]. Medaka ovarian development is classified as asynchronous, meaning a random mixture of oocytes including all developmental stages is present in the ovary without dominant populations. Medaka spawning occurs every day under breeding conditions. Because of these factors and the softness of the chorion in *chg.l −/−* oocytes, it was difficult to collect growing oocytes at the same developmental stage. However, two *chg.l −/−* and two *chg.l +/+* stage VIII oocytes [3] were collected from ovaries of different females and observed via TEM. TEM micrographs revealed the chorion interna of null (*chg.l−/−*) oocytes was less than 1/10 the thickness of that from normal (*chg.l+/+*) oocytes (Fig. 4I and Supplemental Fig. S1).

Medaka oocytes are released from the body of the ovary into the interior ovarian cavity [47, 48] during ovulation. After ovulation, the oocytes pass through the oviduct to the urinogenital orifice [49]. Thus, the *chg.l−/−* oocytes in the present study were likely smashed mechanically during spawning as they passed through the genital duct, producing the string-like material and smashed eggs we observed. Given our findings of the Chg. L protein in control, but not null, female tissues or chorions, and the aforementioned fragility of *chg.l−/−* oocytes, we presume Chg. L (ZI-3) is an essential component of the chorion interna, as it provides support and maintains the structural shape of the oocyte to withstand the pressures exerted against the chorion during spawning. This presumption regarding CHg. L is supported by research into the protein homolog, ZPC, in the mouse ZP. As reported, *ZPC-*KO female mice produced zona-free eggs, ovulated, and exhibited cumulus masses in the oviduct, but few (< 10% of normal) zona-free eggs were recovered [36, 37], and abnormal cumulus-oocyte complexes were observed.

### Chg.L is required for successful oocyte fertilization

The typical pore canals with microvilli were not observed in the chorions of *chg.l−/−* ovarian oocytes from the present study. Instead, abnormally large pore canals that penetrated the follicle cell layer and extended into the oocyte (red arrows in Fig. 4I) were noted. In medaka ovarian oocytes, the pore canals are the structures through which the microvilli pass (Fig. 4C and F). Chg. L also passes through the pore canals, accumulating in developing chorions [17]. Most of the pore canals in the *chg.l−/−* oocytes were compressed and crushed (Fig. 4I). Typical microvilli were not clearly observed in the abnormally large pore canals (Fig. 4H and I). The function of the large pore canals observed in *chg.l−/−* oocyte remains unknown. However, it can be proposed that at least two different functional pore canals exist in medaka ovarian follicles to maintain the physiological condition of growing oocytes and connect the follicle cell layers to the growing oocyte. Despite the lack of Chg. L, the presence of Chg. H was confirmed in the liver and ovary tissue extracts (Fig. 5A) and chorions of *chg.l−/−* females (Fig. 5Bg, Bh, Bk, and Bl). Therefore, the pathway for the gene products of liver-expressed choriogenins may remain intact in the *chg.l−/−* ovary and growing oocytes.

No embryos developed in the *chg.l−/−* oocytes following in vitro fertilization of *chg.l−/−* females. We observed movement of cortical granules and oil droplets but no perivitelline space development. These observations suggested that in fertilization, during the cortical reaction, the cortical granules released the materials from the oocyte between the oocyte plasma membrane and chorion. This process would normally cause the formation of the perivitelline space, but the released materials, such as alveoline [7], transglutaminase [8–10], and lectins [50] — all of which promote hardening of the chorion and form the fertilization membrane that blocks polyspermy — could not interact with the abnormal chorion. This might have resulted in the underdeveloped perivitelline space we observed. Thus, after insemination, polyspermy may have occurred. The micropyle on the surface of the chorion that permits only a single sperm to penetrate the oocyte in teleosts also functions to block polyspermy mechanically [50].

During the early vitellogenic stages of oocyte development in medaka, a micropylar cell differentiates from neighboring granulosa cells. At ovulation, the micropylar cell peels off from the surface of the chorion, and the final form of the micropyle is retained on the surface of the chorion [51]. During oogenesis in the *chg.l−/−* ovary, oocytes may not form a normal micropyle because of the loss of Chg. L (ZI-3). The resulting abnormal micropyle likely allows excess sperm penetration into oocytes and leads to polyspermy.

Further investigation is necessary to confirm this conclusion. One alternative hypothesis is there may be a normal micropyle, but the abnormal structure of the chorion allows excess sperm to penetrate the oocyte resulting in polyspermy. Another alternative hypothesis is that failure to form the perivitelline space during fertilization in *chg.l −/−* inseminated eggs may be a secondary effect unrelated to the physiological function of Chg. L (ZI-3). However, the chorion structure is formed by the coordinated accumulation of various chorion components such as Chgs in medaka.

As shown in the present study, it is obvious the lack of Chg. L (ZI-3) in the chorion resulted in fertilization failure. Additional research is necessary to confirm our conclusion that Chg. L also contributes to blocking polyspermy. In mice, ZPC is known to contribute to the thickness of the ZP [52]. Purified ZP protein domains of trout and mouse ZPC (zp domain: [53–56]) have been shown to form higher-order architectures in the chorion [57, 58]. Given these factors and our findings of a thin chorion interna in *chg.l−/−* oocytes, one of the functions of Chg. L (ZPC in mammals) may be to thicken the chorion during oogenesis. Unlike *ZPC*-KO female mice, *chg.l−/−* female medaka produce oocytes with very thin and soft chorions. In mice, ZPA and ZPB proteins were detected at the surface of the zona-free oocytes [36, 37]. However, as shown in our results, other major components of the medaka chorion, i.e., Chg. H and Chg. Hm (ZPB in mammals), exist as the matrix in the chorion of *chg.l−/−* oocytes (Fig. 5) with unknown structural-support functions.

Based on our cumulative findings in the present study, we propose the following with regard to the chorion and its structural proteins.

1. Chg.L ensures the thickness and strength of the chorion and maintains the oocyte’s structure when placed under stress.
2. Chg.H and Chg. Hm may polymerize individually or each other to construct a fibrous membrane structure. However, without Chg. L, the resultant membrane is too soft to support the oocyte’s structure during oogenesis and spawning. The interaction of Chg. L with Chg. H and Chg. Hm is crucial for the normal chorion architecture.
3. There may exist other unidentified proteins with functions similar to those of Chg. L and its interaction with Chg. H and Chg.Hm. However, in the absence of Chg. L, the expression and production of these unidentified proteins may be insufficient to produce a normal chorion in medaka.
4. Unidentified proteins in medaka, such as the homolog to mammalian ZPA, may be essential and interact with Chg. L, Chg. H, and/or Chg. Hm to contribute to the complete structures of the chorion. This is supported by results obtained in mice, as the murine ZPB homodimer has been shown to cross-link the filaments formed by the ZPA-ZPC matrix [59]. ZPA was shown to interact with ZPC only after the release and incorporation of ZPA and ZPC into the extracellular matrix as ZP [60–62]. The gene homologs to mammalian *ZPA* have not been identified in teleosts.

## Conclusions

The present study suggests Chg. L is the major structural component of the medaka chorion and is essential for fertilization of the oocyte. Thus, gene products of the ovary (e.g., *ZPC 1–5*) are insufficient for maintaining the integrity of the medaka chorion and cannot compensate for the loss of *Chg. L* even though they may have different functions in medaka.

## Supplementary Information

**Additional file 1: Table S1.** The peptide sequences as the antigens for the anti-Chg.L and anti-Chg.H antibodies. The numbers at both ends of the peptide sequences indicate the positions of the predicted amino acid relative to the first methionine in the cDNA with accession ID.

**Additional file 2: Table S2.** The targeted genes for RT-PCR, primers, and the size of the cDNA product and anticipated size of the genomic DNA in Fig. 1.

**Additional file 3: Table S3.** Predicted amino acid sequence of the *chg.l* KO medaka. Each amino acid is represented by a one-letter symbol. The underlined peptide amino acid sequence was used as the antigen to make the anti-Chg.L antibodies. Symbol - Yellow arrow: predicted signal peptide cleavage site.

**Additional file 4: Figure S1.** The thickness the egg envelopes (chorions) in *chg.l−/−* oocytes. The thickness of the chorions in *chg.l+/+* and in *chg.l−/−* females was measured using TEM micrographs. Fifteen different portions of each chorion in two *chg.l+/+* oocytes and two *chg.l−/−* oocytes were selected. The measurements of chorion thickness were analyzed by a student paired t-test (t-Test Calculator: https://www.graphpad.com/quickcalcs/ttest1.cfm). The average chorion thicknesses were 17.65 ± 1.75 μm for oocyte1 (*chg.l+/+*); 16.16 ± 1.41 μm for oocyte2 (*chg.l+/+*); 2.568 ± 1.03 μm for oocyte3 (*chg.l−/−*); and 2.568 ± 1.03 μm for oocyte4 (*chg.l−/−*).

## Data Availability

All data generated or analyzed during this study are included in this published article [and its supplementary information files].

## Abbreviations

Chg: Choriogenin
ZP: Zona pellucida
*chg.l*−/−: Homozygous *Choriogenin L* knock out
*chg.l*+/−: Heterozygous *Choriogenin L* knock out
*chg.l+/+*: A wild-type Cab medaka
BTTBSG: 2% BSA-TBS containing 0.05% Tween-20 and 2% preimmune goat serum
TBS: Tris buffered saline
