# Supplementary material for: Targeted deletion of liver-expressed Choriogenin L results in the production of soft eggs and infertility in medaka, Oryzias latipes
Source: Zoological Lett. 2022 Jan 4;8:1. doi: 10.1186/s40851-021-00185-9 (PMC8729012; doi:10.1186/s40851-021-00185-9)
Supplement: Supplementary file 1 — Additional file 1: Table S1. The peptide sequences as the antigens for the anti-Chg.L and anti-Chg.H antibodies. The numbers at both ends of the peptide sequences indicate the positions of the predicted amino acid relative to the first methionine in the cDNA with accession ID. [file 40851_2021_185_MOESM1_ESM.pdf]

Genotype	Amino Acid sequence	Accession ID
Choriogenin L	26-GKPSYPPTGSKTPQ(C)-39	NM_001104803
Choriogenin H	207-(C)QYPSKPQDPGKNPN-220	NM_001104807
