# Supplementary material for: Targeted deletion of liver-expressed Choriogenin L results in the production of soft eggs and infertility in medaka, Oryzias latipes
Source: Zoological Lett. 2022 Jan 4;8:1. doi: 10.1186/s40851-021-00185-9 (PMC8729012; doi:10.1186/s40851-021-00185-9)
Supplement: Supplementary file 2 — Additional file 2: Table S2. The targeted genes for RT-PCR, primers, and the size of the cDNA product and anticipated size of the genomic DNA in Fig. 1. [file 40851_2021_185_MOESM2_ESM.pdf]

Position	Fw primer	Rv primer	Predicted PCR size (bp)	gDNA
Chromosome 6: 20,586,980-20,587,519	GTGCTGGCCCTGCTTGACGGC<br>Chg.L FW1	GAAGGTGTAGACCAGGGAGTC<br>Chg.L RV2	423	540
Chromosome 6: 20,586,980-20,587,455	CAACAGGGAGTAAAACGCCTCA<br>Chg.L FW2-del	GAAGGTGTAGACCAGGGAGTC<br>Chg.L RV2	359	476
Chromosome 6: 21,387,202-21,387,443	GACATCTCTCCTTCTGCATGTGA<br>Chg.H Ex1 FW1	TCCAGGTCAATGTGAGGTAAGGTG<br>Chg.H Ex2 RV1	152	242
Chromosome 6: 20,591,285-20,591,585	ATGGACACACTTGCTACTATGGCA<br>Chg.Hm Ex1 FW1	TAGTGCCACAAGAGGTGACTGGA<br>Chg.Hm Ex2 RV1	207	301
Chromosome 6: 20,591,285-20,591,749	ATGGACACACTTGCTACTATGGCA<br>Chg.Hm Ex1 FW1	ATGGCTCCACGAGGTCCAATAGC<br>Chg.Hm Ex3 RV1	292	292
