# Supplementary material for: Targeted deletion of liver-expressed Choriogenin L results in the production of soft eggs and infertility in medaka, Oryzias latipes
Source: Zoological Lett. 2022 Jan 4;8:1. doi: 10.1186/s40851-021-00185-9 (PMC8729012; doi:10.1186/s40851-021-00185-9)
Supplement: Supplementary file 3 — Additional file 3: Table S3. Predicted amino acid sequence of the chg.l KO medaka. Each amino acid is represented by a one-letter symbol. The underlined peptide amino acid sequence was used as the antigen to make the anti-Chg.L antibodies. Symbol - Yellow arrow: predicted signal peptide cleavage site. [file 40851_2021_185_MOESM3_ESM.pdf]

Genotype	Amino Acid sequence
Wild type	MMKFTAVCLVVLALLDGFCDAQHNYGKPSYPPTGSKTPQDPTQQKQLHEKELTWKYPADPQPEAKPV<br>VPFEQRYPVPAATVAVECREDLAHVEAKKDLFGIGQFIDPADLTLGTCPPSAEDPAAQVLIFESPLQ<br>NCGSVLTMTEDSLVYTFTLNYNPKPLGSAPVVRTSQAVVIVECHYPRKHNVSSLALDPLWVPFSAAK<br>MAEEFLYFTLKLTTDDFQFERPSYQYFLGDLIHIEATVKQYFHVPLRVYVDRCVATLSPDANSSPSY<br>AFIDNYGCLLDGRITGSDSKFVSRPAENKLDFQLEAFRFQGADSGMIYITCHLKATSAAYPLDAEHR<br>ACSYIQGWKEVSGADPICASCESGGFEVHANAVVSHGTSTLSGGGHGTGKPSDPSRKTREAAKTEVL<br>EWEGDVTLGPIPIEERRV*
Mutant (delta-14)	MMKFTAVCLVVLALLDGFCDAQHNYGKPSYPPTGRSHPAKAVA*
