# Supplementary figures and images for: Targeted deletion of liver-expressed Choriogenin L results in the production of soft eggs and infertility in medaka, Oryzias latipes
Source: Zoological Lett. 2022 Jan 4;8:1. doi: 10.1186/s40851-021-00185-9 (PMC8729012; doi:10.1186/s40851-021-00185-9)

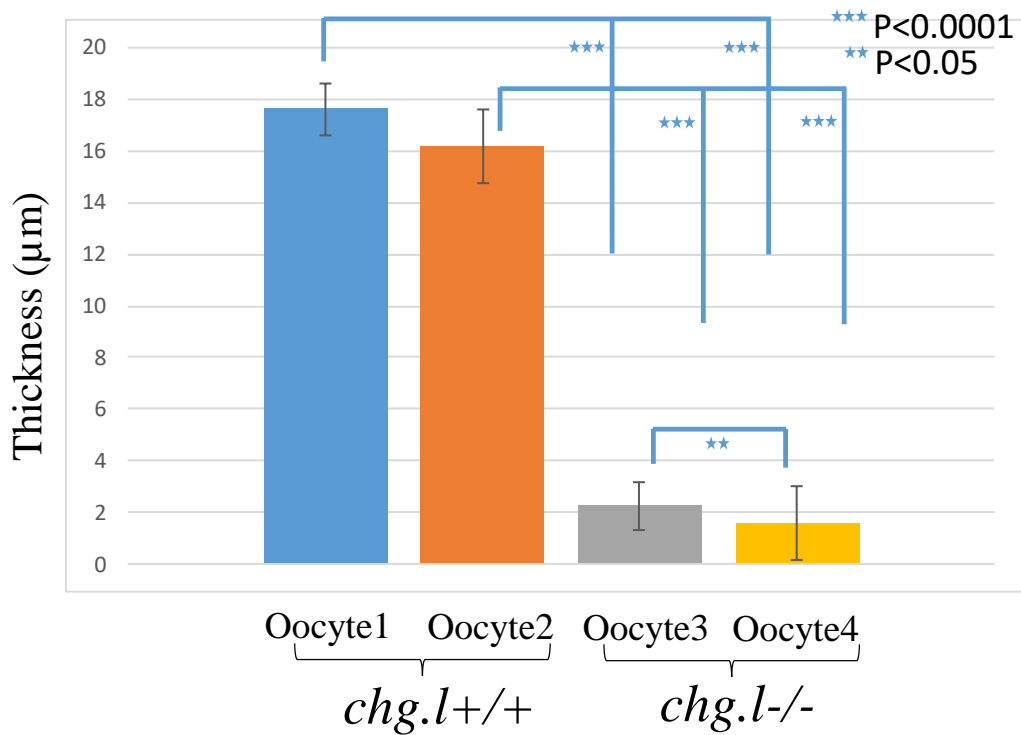

Supplement: Supplementary file 4 — Additional file 4: Figure S1. The thickness the egg envelopes (chorions) in chg.l−/− oocytes. The thickness of the chorions in chg.l+/+ and in chg.l−/− females was measured using TEM micrographs. Fifteen different portions of each chorion in two chg.l+/+ oocytes and two chg.l−/− oocytes were selected. The measurements of chorion thickness were analyzed by a student paired t-test (t-Test Calculator: https://www.graphpad.com/quickcalcs/ttest1.cfm). The average chorion thicknesses were 17.65 ± 1.75 μm for oocyte1 (chg.l+/+); 16.16 ± 1.41 μm for oocyte2 (chg.l+/+); 2.568 ± 1.03 μm for oocyte3 (chg.l−/−); and 2.568 ± 1.03 μm for oocyte4 (chg.l−/−). [file 40851_2021_185_MOESM4_ESM.pdf]
